# A Rocking-chair Rechargeable Seawater Battery

**DOI:** 10.34133/research.0461

**Published:** 2024-08-27

**Authors:** Jialong Wu, Yongshuo Zheng, Pengfei Zhang, Xiaoshuang Rao, Zhenyu Zhang, Jin-Ming Wu, Wei Wen

**Affiliations:** ^1^ Collaborative Innovation Center of Ecological Civilization, School of Mechanical and Electrical Engineering, Hainan University, Haikou 570228, China.; ^2^ State Key Laboratory of High-performance Precision Manufacturing, Dalian University of Technology, Dalian 116024, China.; ^3^ State Key Laboratory of Silicon and Advanced Semiconductor Materials, School of Materials Science and Engineering, Zhejiang University, Hangzhou 310027, China.

## Abstract

Seawater batteries are attracting continuous attention because seawater as an electrolyte is inexhaustible, eco-friendly, and free of charge. However, the rechargeable seawater batteries developed nowadays show poor reversibility and short cycle life, due to the very limited electrode materials and complicated yet inappropriate working mechanism. Here, we propose a rechargeable seawater battery that works through a rocking-chair mechanism encountered in commercial lithium ion batteries, enabled by intercalation-type inorganic electrode materials of open-framework-type cathode and Na-ion conducting membrane-type anode. The rechargeable seawater battery achieves a high specific energy of 80.0 Wh/kg at 1,226.9 W/kg and a high specific power of 7,495.0 W/kg at 23.7 Wh/kg. Additionally, it exhibits excellent cycling stability, retaining 66.3% of its capacity over 1,000 cycles. This work represents a promising avenue for developing sustainable aqueous batteries with low costs.

## Introduction

The variability of renewable energy sources like solar, wind, and geothermal necessitates dependable electrochemical energy storage systems for large-scale grid storage [1–6]. For grid-scale stationary applications, affordability and safety are crucial [7,8]. Among a variety of rechargeable batteries, aqueous sodium ion batteries are considered promising options for large-scale energy storage, owing to their affordability, nontoxicity, inherent safety, and high abundance [9–13], which utilize Na_2_SO_4_, NaCl, NaNO_3_, or NaClO_4_ aqueous solutions as electrolytes. The seawater electrolyte can effectively address the sustainable challenges in rechargeable batteries requiring harmless and earth-abundant electrolytes and electrodes [14], because of its merits of being inexhaustible, eco-friendly, and free of charge.

The mature seawater batteries early developed are primary batteries [15–17], where Mg or Al metals serve as anodes, and AgCl or dissolved oxygen serves as cathodes. Rechargeable half-seawater batteries have been proposed recently [18–22], which use air cathodes working in seawater; however, the anodes (typically Na metal or hard carbon) can only work in conventional nonaqueous electrolytes, making it combustible and expensive. It is noted that supercapacitors can directly use seawater as the only electrolyte [23–25], but their specific energies are too low. For example, the specific energy of the supercapacitor based on an activated carbon is only 7.7 Wh/kg [23]. Rechargeable full-seawater batteries (RSWBs), which employ seawater as both catholyte and anolyte, are rarely reported due to the limited electrodes. We recently developed a lattice-expansion strategy to enable anatase TiO_2_ as a high-capacity and high-rate anode for RSWBs [26]. High specific energies can be obtained in this RSWB; however, the energy efficiency is low and the cycling stability is still unsatisfactory, because an air cathode is required [26]. It is thus urgent yet challenging to explore new low-cost electrode materials working with more feasible modes.

Compared to conventional aqueous sodium ion batteries, the intercalation of Na^+^ may be interfered or hindered by other ions in RSWBs because of complex compositions of natural seawater. Among electrode options for RSWBs, it is expected that the electrode materials should meet one of the following 2 criteria: (a) they could intercalate multiple ions; (b) they can intercalate a kind of ion from seawater and simultaneously exhibit strong anti-interference ability for other ions. In this regard, open-framework crystal structures with large interval space, like Prussian blue analogs (PBAs), can accommodate multiple ions with a good structural deformation tolerance [27–29]. We also note that, in rechargeable half-seawater batteries, a Na-ion conducting membrane (NASICON) is employed to separate the seawater catholyte and the nonaqueous electrolytes, while allowing only Na^+^ transport between the 2 electrolytes [18–22]. The good stability of NASICON-type materials in seawater hints their strong ion anti-interference ability and mechanical robustness, which makes them possible electrode materials for RSWBs.

**Fig. 1.**
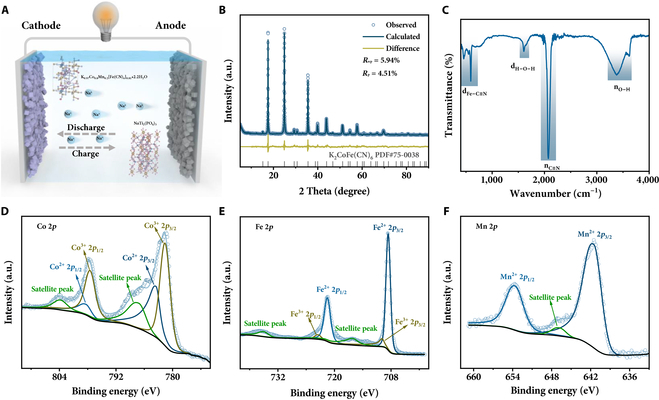
Schematic diagram for rocking-chair rechargeable seawater battery and spectral characterization of cathode material. (A) The schematic illustration of K_0.97_Co_0.8_Mn_0.2_[Fe(CN)_6_]_0.81_•2.2H_2_O//NaTi_2_(PO_4_)_3_/C full rechargeable seawater battery. (B) Rietveld refined XRD pattern, (C) FTIR spectrum, and (D to F) XPS spectra of Co 2*p*, Fe 2*p*, and Mn 2*p* for K_0.97_Co_0.8_Mn_0.2_[Fe(CN)_6_]_0.81_•2.2H_2_O.

In view of aforementioned aspects, we propose a full RSWB constructed with a Mn-substituted Co-rich K_0.97_Co_0.8_Mn_0.2_[Fe(CN)_6_]_0.81_•2.2H_2_O cathode and NASICON-type NaTi_2_(PO_4_)_3_/C anode, which can work in natural seawater electrolytes (Fig. 1A). The battery operates on the basis of a rocking-chair mechanism by using intercalation-type inorganic electrode materials, which is just the same as commercialized lithium-ion batteries work. The RSWB exhibits a high specific energy of 80.0 Wh/kg at 1,226.9 W/kg and a high specific power of 7,495.0 W/kg at 23.7 Wh/kg. Additionally, it exhibits excellent cycling stability, retaining 66.3% of its capacity over 1,000 cycles. This work may promote the development of sustainable and low-cost aqueous batteries.

## Results and Discussion

### Open-framework-type cathode

Numerous PBA cathodes have been applied in aqueous batteries, yet none of them demonstrate high capacities in RSWBs. It is reported that Mn-rich or Co-rich PBAs show relatively high capacities in aqueous sodium or potassium ion batteries [29,30]. In this investigation, Co-rich PBA was utilized as cathode for RSWBs, and Mn-substitution strategy was further applied to improve its capacities. The cathode material was synthesized at room temperature using a coprecipitation method, employing low-cost raw materials of K_3_Fe(CN)_6_, cobalt chloride, manganese chloride, and sodium citrate. All of the Bragg diffraction peaks in the x-ray diffraction (XRD) pattern of the optimized sample, with a Co/Mn atomic ratio of 0.8/0.2 and the addition of sodium citrate, can be well indexed to orthogonal phase PBA (Fig. 1B) [31]. The cell constants *a*, *b*, and *c* and cell volume *V* were determined to be 10.10, 7.14, and 7.14 Å and 514.58 Å^3^ by Rietveld refinement with a robust fit. Figure 1C displays the Fourier transform infrared spectrum (FTIR), which clearly shows the characteristic absorption peaks corresponding to the Fe–C≡N, H–O–H, C≡N, and O–H vibrations [32], further verifying the successful synthesis of the PBA. The atomic ratio of K/Co/Mn/Fe, determined by inductively coupled plasma optical emission spectrometry, is found to be 0.97/0.8/0.2/0.81. The thermogravimetric curve (Fig. S1), indicates a weight loss of 12.9%, corresponding to 2.2 H_2_O in this PBA. Hence, the chemical formula of the prepared sample can be expressed as K_0.97_Co_0.8_Mn_0.2_[Fe(CN)_6_]_0.81_•2.2H_2_O.

To investigate the valence states of all the elements in the sample, we conducted x-ray photoelectron spectroscopy (XPS). As depicted in Fig. 1D to F and Fig. S2, it verified the existence of K, Co, Mn, Fe, C, N, and O elements without any other impurities. As shown in Fig. 1D, the binding energies of 783.5 and 798.5 eV correspond to the Co^2+^ 2*p*_3/2_ and Co^2+^ 2*p*_1/2_ peaks, respectively, while the Co^3+^ 2*p*_3/2_ and Co^3+^ 2*p*_1/2_ peaks were also observed around 781.6 and 797.5 eV, respectively [33,34]. The Fe 2*p* edge peaks (Fig. 1E) at 708.5 and 721.6 eV correspond to 2*p*_3/2_ and 2*p*_1/2_ spins of Fe^2+^, while those at 709.9 and 723.5 eV can be ascribed to 2*p*_3/2_ and 2*p*_1/2_ spins of Fe^3+^ [34]. The Mn 2*p* spectrum were successfully deconvoluted into 3 peaks representing the characteristic Mn^2+^ 2*p*_3/2_ (641.5 eV), Mn^2+^ 2*p*_1/2_ (653.6 eV), and a satellite peak, respectively (Fig. 1F) [35]. The fitted C 1*s* spectrum reveals 3 main peaks at 284.8, 285.2, and 287.7 eV, which correspond to C–C, C≡N, and N–C=O, respectively (Fig. S2B) [36]. Thus, the Co and Fe elements in the sample are in mixed valence states of +2 and +3, and the Mn valence state is +2.

**Fig. 2.**
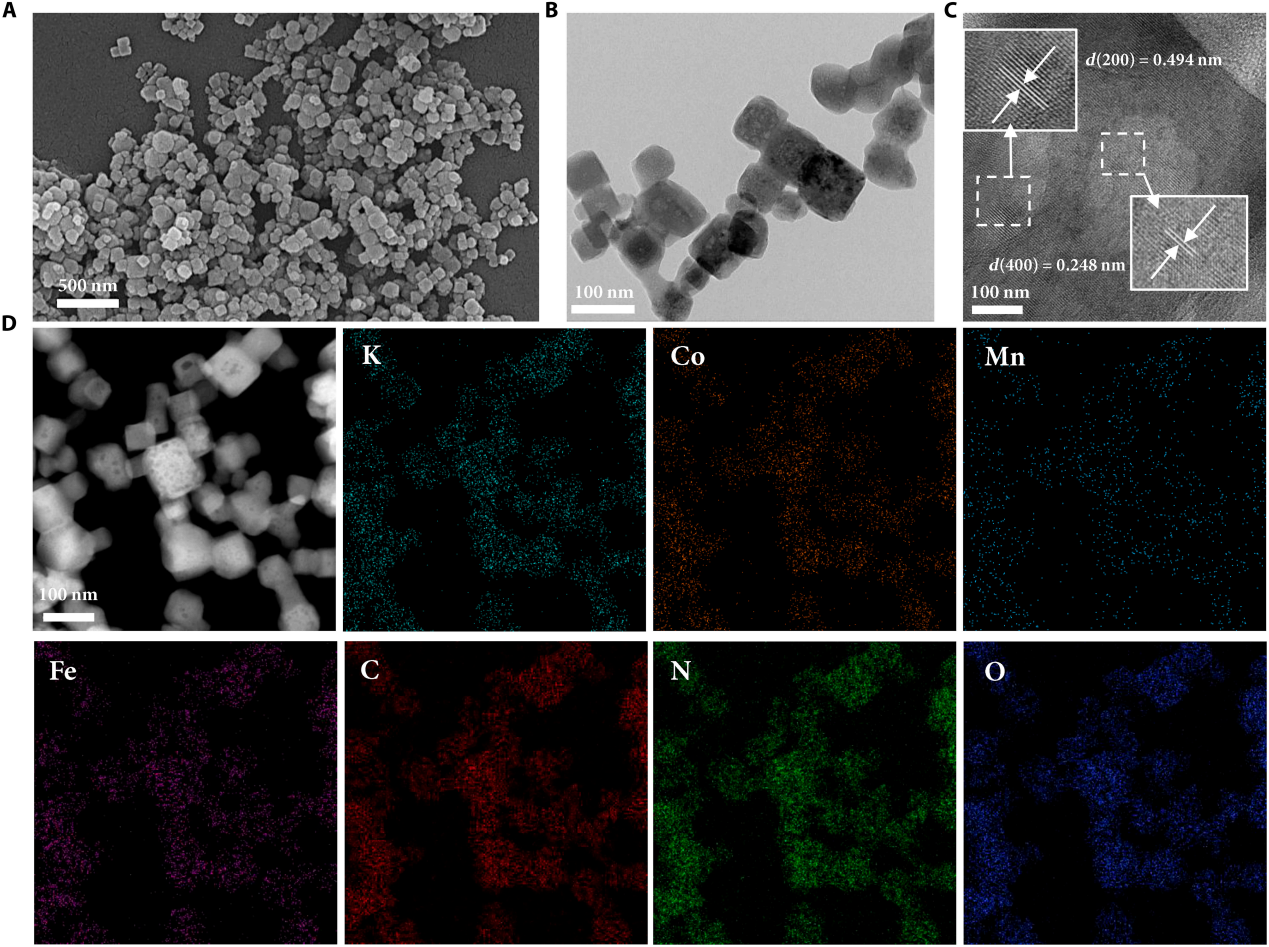
Structural characterization of cathode material. (A) Scanning electron microscopy image, (B) TEM image, (C) HRTEM image, and (D) EDX mapping of K_0.97_Co_0.8_Mn_0.2_[Fe(CN)_6_]_0.81_•2.2H_2_O.

The scanning electron microscopy and transmission electron microscopy (TEM) images show that the K_0.97_Co_0.8_Mn_0.2_[Fe(CN)_6_]_0.81_•2.2H_2_O cathode exhibits a regular cubic shape (Fig. 2A and B). In the high-resolution TEM (HRTEM) image (Fig. 2C), the clear lattice spacing of 0.494 and 0.248 nm are ascribed to the (200) and (400) crystal planes of the K_0.97_Co_0.8_Mn_0.2_[Fe(CN)_6_]_0.81_•2.2H_2_O. The energy-dispersive x-ray spectroscopy (EDX) mappings (Fig. 2D and Fig. S3) demonstrate the even distribution of Fe, Mn, Co, K, N, C, and O over the sample. The specific surface area is determined to be 125.5 m^2^/g, and the average pore diameter is 18.3 nm (Fig. S4).

**Fig. 3.**
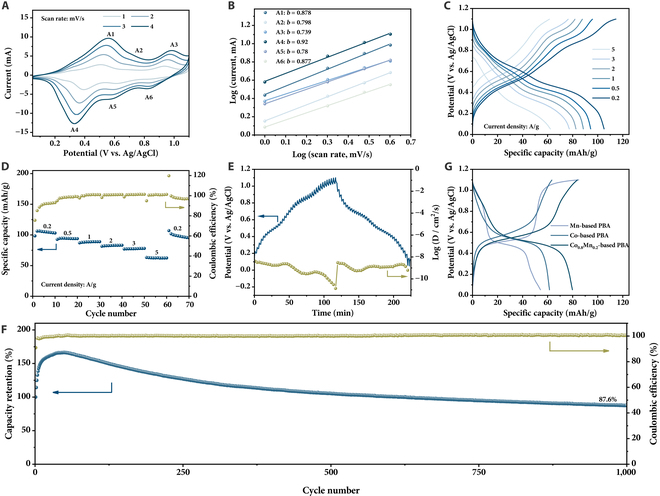
Electrochemical performance of cathode material. (A) CV profiles at different scan rates, (B) the corresponding log(*i*)–log(*v*) curves for the peaks from A1 to A6, (C) GCD curves at different current densities, (D) rate performance, (E) galvanostatic intermittent titration technique response and ion diffusion coefficients, and (F) cycling stability at 3 A/g for K_0.97_Co_0.8_Mn_0.2_[Fe(CN)_6_]_0.81_•2.2H_2_O. (G) GCD curves at 0.5 A/g of Mn-based PBA, Co-based PBA, and Co_0.8_Mn_0.2_ PBA samples. The samples in (G) were synthesized without the addition of sodium citrate.

To evaluate the electrochemical performance of the K_0.97_Co_0.8_Mn_0.2_[Fe(CN)_6_]_0.81_•2.2H_2_O in natural seawater, electrochemical tests were carried out in a standard 3-electrode system. The ion concentrations of Na, K, Ca, and Mg in natural seawater were determined to be 10,147, 440, 407, and 120 mg/l by inductively coupled plasma optical emission spectrometry, respectively. According to the cyclic voltammogram (CV) curves depicted in Fig. 3A, 3 pairs of redox peaks can be observed, suggesting 3 active sites for the energy storage in the K_0.97_Co_0.8_Mn_0.2_[Fe(CN)_6_]_0.81_•2.2H_2_O. The relationship of the current (*i*) and the scan rate (*v*) follows a power law of *i* = *av^b^*. The *b* value equal to 1.0 represents surface-controlled electrochemical reaction, while *b* = 0.5 means that the electrochemical reaction is controlled by semi-infinite diffusion. The *b* values of A1 to A6 are 0.878, 0.798, 0.739, 0.92, 0.78, and 0.877, respectively (Fig. 3B). The high *b* values (0.73 to 0.92) suggest a high pseudocapacitive contribution, because the open-framework crystal structure allows fast ion transport.

The electrochemical performance of the K_0.97_Co_0.8_Mn_0.2_[Fe(CN)_6_]_0.81_•2.2H_2_O was further evaluated by galvanostatic charge–discharge (GCD) (Fig. 3C). Consistent with the above CV results, 3 discharge plateaus are observed. At the current densities of 0.2, 0.5, 1, 2, 3, and 5 A/g, the specific capacities of K_0.97_Co_0.8_Mn_0.2_[Fe(CN)_6_]_0.81_•2.2H_2_O are 105.2, 94.5, 88.6, 82.7, 77.4, and 62.4 mAh/g, respectively (Fig. 3C and D). The discharge capacity recovers to 101.78 mAh/g when the current density was reduced to 0.2 A/g, indicating excellent rate capacity and high reversibility. To understand the kinetic properties as a function of the charge/discharge depth, the galvanostatic intermittent titration technique was subsequently utilized (Fig. 3E). The diffusion coefficient is from 10^−11^ to 10^−9^ cm^2^/s in the charge process, while it maintains a high level (~10^−9^ cm^2^/s) during the discharge process, conducive to the outstanding rate capability. Additionally, the K_0.97_Co_0.8_Mn_0.2_[Fe(CN)_6_]_0.81_•2.2H_2_O shows attractive cycling stability, with 87.6% capacity retention over 1,000 cycles at 3 A/g (Fig. 3F). The initial gradual rise in capacity can be ascribed to the activation process, which occurs as the electrolyte gradually infiltrates the porous electrode [37].

The capacity of the Mn-substituted Co-based PBA surpasses those of both the Mn-based PBA and Co-based PBA (Fig. 3G). The optimized atomic ratio for Co/Mn was 0.8/0.2 for simultaneously achieving high capacity and good cycling stability (Fig. 3 and Fig. S5). Although the crystalline water contents in the Mn-based (27.4%), Co-based (29.4%), and Co_0.8_Mn_0.2_-based (27.6%) PBAs are close (Fig. S6), the morphology and size are quite different (Fig. S7). The Mn-based PBA exhibits a regular cube morphology, while its size is in micrometer scale, which leads to the low specific capacity. On the contrary, although the Co-based PBA possesses nanoscale particle size, its appearance is irregular. The Co_0.8_Mn_0.2_-based PBA combines the advantages of small particle size and high crystal quality with a regular shape, contributing to the higher specific capacity. The observed capacity decay in Mn-based PBA may originate from the structural deformation of Mn-N_6_ octahedra due to Jahn–Teller distortion during Na^+^/K^+^ ion insertion/extraction, which leads to the dissolution of active materials in the electrolyte [38]. Introducing cobalt into Mn-based PBAs is effective in alleviating Jahn–Teller distortion and maintaining the stability of the crystal structure [39,40], thereby extending the cycle life. Furthermore, the sodium citrate utilized in our synthesis process also contributes to the improvement in the cycling stability (Fig. 3F and Fig. S5), owing to its capability for increasing crystal quality [41]. It was found that the presence of sodium citrate during the preparation process can also increase the potassium content and reduce the crystal water content in the obtained PBAs (K_0.97_Co_0.8_Mn_0.2_[Fe(CN)_6_]_0.81_•2.2H_2_O versus K_0.08_Co_0.8_Mn_0.2_[Fe(CN)_6_]_0.7_•4.45H_2_O) due to its reducibility. Therefore, the doping strategy and the crystal quality regulation developed herein contribute to the enhanced specific capacity and cycling stability.

**Fig. 4.**
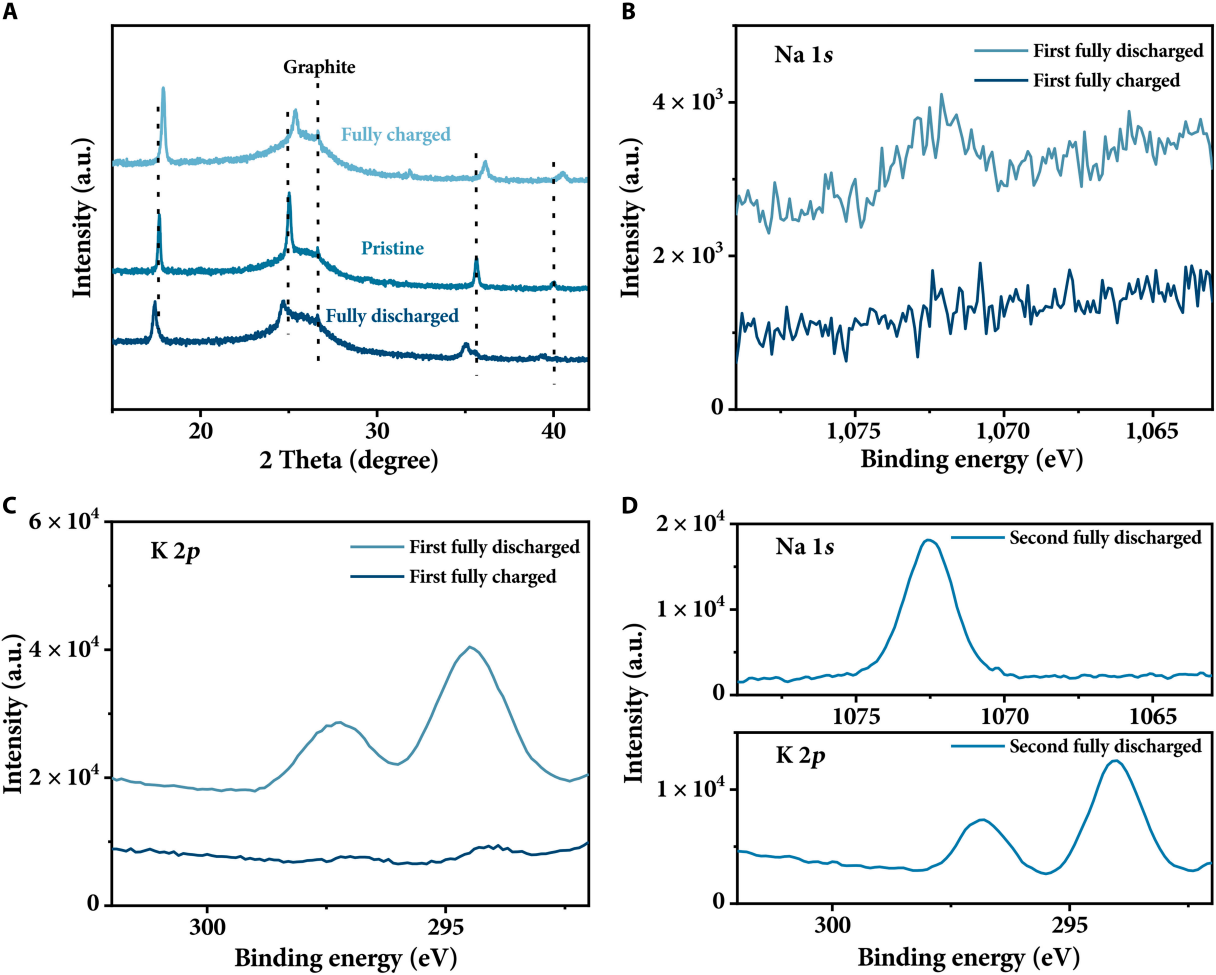
Comparison of structure and composition for cathode material at fully charged and discharged states. (A) XRD patterns of K_0.97_Co_0.8_Mn_0.2_[Fe(CN)_6_]_0.81_•2.2H_2_O at pristine and fully charged/discharged states. (B and C) Na 1*s* and K 2*p* XPS spectra of K_0.97_Co_0.8_Mn_0.2_[Fe(CN)_6_]_0.81_•2.2H_2_O at the first fully discharged/charged states. (D) High-resolution Na 1*s* and K 2*p* XPS spectra of K_0.97_Co_0.8_Mn_0.2_[Fe(CN)_6_]_0.81_•2.2H_2_O at the fully discharged/charged states in the second cycle. The fully discharged state was obtained by discharging the cathode to 0.05 V, while the fully charged state corresponds to 1.1 V.

The optimized PBA at fully charged and discharged states were further characterized by XRD, XPS, and EDX for comparative analysis. The peak near 27° in the XRD patterns (Fig. 4A) originates from the carbon cloth current collector and remains unchanged throughout the charge/discharge process. Compared with the discharged state, the Bragg diffraction peaks of sample at charged state shift toward higher angles. This lattice contraction when charged from 0.05 to 1.1 V is ascribed to the cation deintercalation reaction. After the first fully discharged (cation intercalation) process, the XPS peak intensities assigned to Na^+^ and K^+^ obviously increase compared to the first fully charged state (Fig. 4B and C). Subsequently, an increase in Na^+^ and a decrease in K^+^ can be found in the second discharge state (Fig. 4B to D). The Na/K ratio in discharge state increase from 0.78 in the 1st cycle to 2.4 in the 500th cycle (Table S1), indicating that the main charge carrier is Na^+^. The electrochemical properties in different aqueous electrolytes were also compared to further analyze the types of key charge carriers. The capacity follows the order of Na^+^>K^+^>Mg^2+^>Ca^2+^ (86.6, 58.5, 25.2, and 22.8 mAh/g, respectively), as shown in Fig. S8. Although the leading charge carrier is Na^+^, we used K_3_Fe(CN)_6_ instead of Na_3_Fe(CN)_6_ in the cathode preparation, owing to the much lower price of the former.

### NASICON-type anode

NASICON-type NaTi_2_(PO_4_)_3_ consists of an open 3D framework of TiO_6_ octahedra and PO_4_ tetrahedra, which may have potential applications in RSWBs. Hydrothermal process was used to synthesize NaTi_2_(PO_4_)_3_, which was subsequently coated with a carbon layer to enhance both its conductivity and stability. As confirmed by the XRD pattern (Fig. S9A), well-crystallized NASICON-type NaTi_2_(PO_4_)_3_ was successfully prepared. The FTIR spectrum distinctly exhibits characteristic absorption peaks corresponding to P–O and Ti–O bond vibrations (Fig. S9B) [42,43]. Raman spectroscopy further confirms the presence of Ti–O and P–O bonds and carbon coating (Fig. S9C) [43]. The weight percentage of the carbon coating in the NaTi_2_(PO_4_)_3_/C is approximately 1.7 wt.%, quantified from thermogravimetric analysis in air (Fig. S9D). As shown in Fig. S10A and B, the NaTi_2_(PO_4_)_3_/C demonstrates a polyhedron shape with the size of hundreds of nanometers. The parallel lattice fringes in the HRTEM image (Fig. S10C) are assigned to the (113) crystallographic planes of NaTi_2_(PO_4_)_3_. As shown in Fig. S10C, the carbon layer on the surface of the NaTi_2_(PO_4_)_3_ is ca. 4.3 nm in thickness. Additionally, the compositions are uniform in the as-prepared NaTi_2_(PO_4_)_3_/C (Fig. S10D). The average pore diameter is 28.7 nm, and the specific surface area is 13.2 m^2^/g (Fig. S11). The chemical compositions of anode materials were analyzed by XPS (Fig. S12). The existence of Ti, P, Na, O, and C elements was revealed by the survey XPS spectrum of anode materials. The Ti 2*p* spectrum contains 2 peaks, corresponding to the characteristic Ti ^4+^ 2*p*_3/2_ (460.7 eV) and Ti ^4+^ 2*p*_1/2_ (466.4 eV) [44]. The O 1*s* spectrum can be deconvoluted into 3 components corresponding to the P–O–H (531.9 eV), O–C (532.7 eV), and P–O–Na or P–O–Ti or P=O (531.4 eV) [45]. The fitted C 1*s* spectrum shows 3 main peaks at 284.8, 286.3, and 288.6 eV, which correspond to C–C/C=C, C–O, and O–C=O, respectively [46,47].

**Fig. 5.**
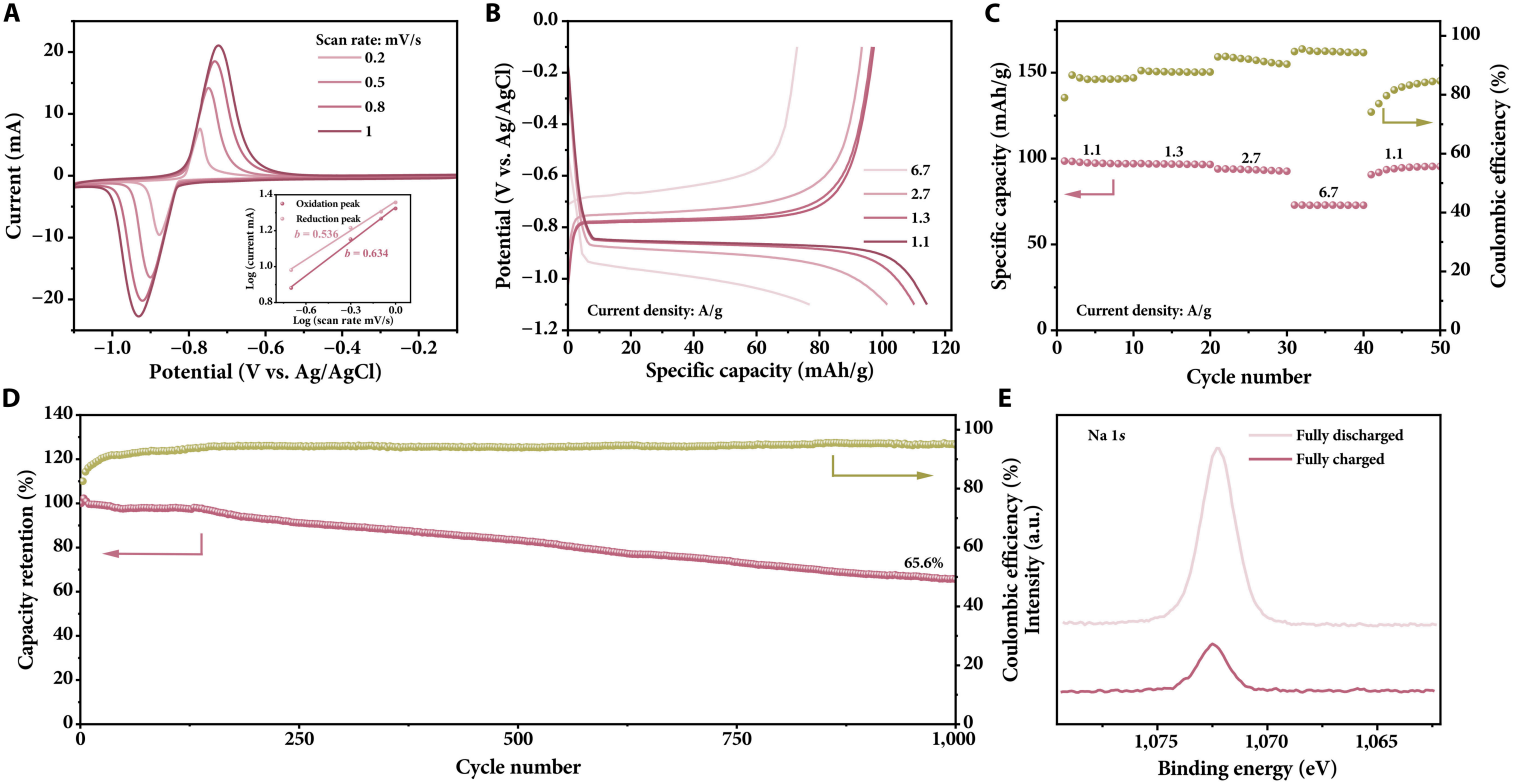
Electrochemical performance of anode material. (A) CV profiles at different scan rates, (B) GCD curves at different current densities, (C) rate performance, and (D) cycling stability at 1.1 A/g for NaTi_2_(PO_4_)_3_/C. The inset in (A) shows the relationship between the peak currents and scan rates. (E) High-resolution Na 1*s* XPS spectra of NaTi_2_(PO_4_)_3_/C in the fully discharged and charged states.

CV measurements (Fig. 5A) were performed at different sweep rates spanning from 0.2 to 1.0 mV/s. A pair of sharp peaks can be clearly observed in the CV curves. The CV curve shapes stay consistent across different scan rates, with only a slight shift in the potentials of the redox peaks. The *b* values of oxidation and reduction peaks are 0.63 and 0.54, respectively, slightly higher than 0.5, which indicate that Na^+^ storage in NaTi_2_(PO_4_)_3_/C is predominantly diffusion-controlled intercalation. Consistent with the CV results, clear charge/discharge plateaus corresponding to the Na^+^ intercalation/deintercalation can be observed (Fig. 5B). The specific capacities of NaTi_2_(PO_4_)_3_/C are 97.3, 96.7, 93.5, and 72.9 mAh/g at 1.1, 1.3, 2.7, and 6.7 A/g, respectively (Fig. 5C). The specific capacity recovers to 90.6 mAh/g when the current density was reduced to 1.1 A/g, indicating the excellent rate capacity and high reversibility. The fluctuation of coulombic efficiency in Fig. 5C may be attributed to the side reactions of the anode material, such as hydrogen evolution reactions (Fig. S13). At lower current densities, the more severe hydrogen evolution reaction results in the lower coulombic efficiency. The cycling stability evaluation of the NaTi_2_(PO_4_)_3_/C at 2.7 A/g reveals a high capacity retention of 65.6% after 1,000 cycles (Fig. 5D).

Upon comparing the GCD profiles in different electrolytes with identical cation concentrations, it was found that Na^+^ is the key charge carrier for the energy storage of the NaTi_2_(PO_4_)_3_/C (Fig. S14). The specific capacities in 0.5 M NaCl, KCl, MgCl_2_, and CaCl_2_ are 73.8, 8.3, 6.8, and 6.6 mAh/g, respectively. Ex situ XRD and XPS results were obtained at fully charged and discharged states of the NaTi_2_(PO_4_)_3_/C to further elucidate the Na^+^ storage mechanism. No obvious peak changes occur in the charge/discharge process, as illustrated in Fig. S15. This observation indicates that the Na^+^ intercalation/deintercalation process occurs without phase transformation, contributing to the good structural stability. After the first discharge (Na^+^ insertion) process, the intensity for the Na 1*s* peak in the XPS spectrum sharply increases compared to that in the fully charged state (Fig. 5E and Fig. S16). In addition, the EDX analysis for pristine, fully discharged, and fully charged samples reveals the changes in the content of Na^+^ in the anode throughout the charge/discharge processes, thereby supporting the inference that the energy storage is primarily governed by the Na^+^ intercalation/deintercalation process (Table S2).

### Full battery

**Fig. 6.**
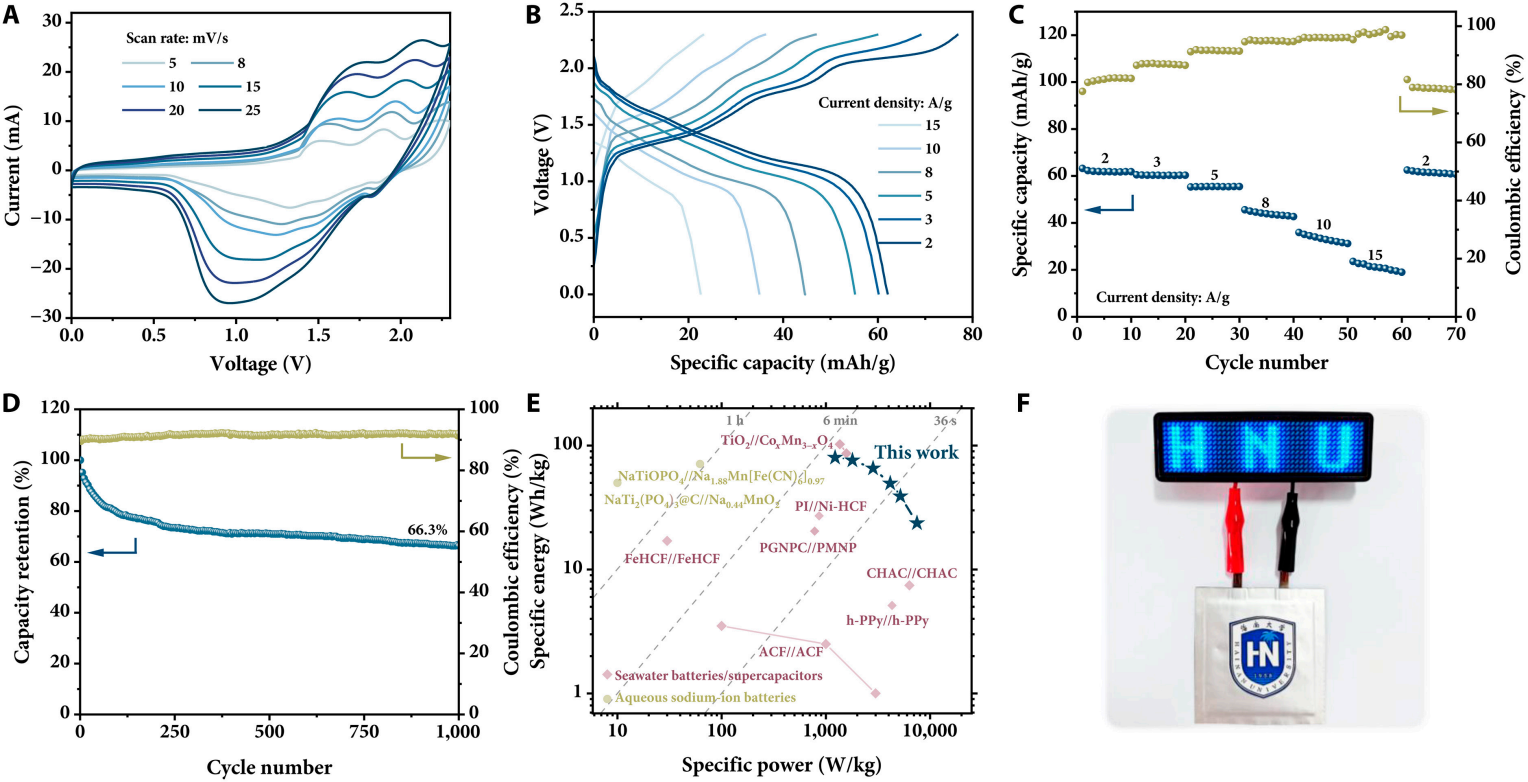
Electrochemical performance of full cell. (A) CV curves at different scan rates, (B) GCD profiles at different current densities, (C) rate performance, and (D) cycling stability at 5 A/g. (E) Ragone plots for the state-of-art rechargeable seawater batteries and aqueous Na^+^ batteries. (F) An optical photograph of the assembled RSWB pouch cell for powering a light-emitting diode display screen.

In our full cell design, the specific capacity of the K_0.97_Co_0.8_Mn_0.2_[Fe(CN)_6_]_0.81_•2.2H_2_O is close to that of the NaTi_2_(PO_4_)_3_/C (Figs. 3D and 5C), which is beneficial for achieving high specific capacities for a full battery. The mass ratio of the NaTi_2_(PO_4_)_3_/C and K_0.97_Co_0.8_Mn_0.2_[Fe(CN)_6_]_0.81_•2.2H_2_O is set as 1.1:1, and natural seawater was used as electrolyte. A full battery was operated in 0 to 2.3 V. Three pairs of oxidation/reduction peaks in the CV curves are observed (Fig. 6A), which is similar to the cathode (Fig. 3A). At 2 A/g (based on the active material of the K_0.97_Co_0.8_Mn_0.2_[Fe(CN)_6_]_0.81_•2.2H_2_O), the median discharge voltage is ca. 1.288 V (Fig. 6B). The specific capacities (based on the active materials of the K_0.97_Co_0.8_Mn_0.2_[Fe(CN)_6_]_0.81_•2.2H_2_O and the NaTi_2_(PO_4_)_3_/C) of the full battery are 62.1, 60.2, 55.2, 44.8, 35, and 23.5 mAh/g at 2, 3, 5, 8, 10, and 15 A/g, respectively (Fig. 6C). The fluctuation in the coulombic efficiency of the full cell in Fig. 6C can be also ascribed to the side reactions of the electrode materials. The full battery exhibits excellent cycling stability with 66.3% capacity retention over 1,000 cycles at 5 A/g (Fig. 6D). The cathode material demonstrates a relatively higher specific capacity in 0.5 M KCl aqueous electrolyte (Fig. S8B), while the anode material exhibits a very low specific capacity in this electrolyte (Fig. S14B and Table S2). Based on the rocking chair mechanism and the very low concentration of K^+^ in seawater (the Na/K atomic ratio in the seawater is 39/1), the main carrier in the full cell is Na^+^ instead of K^+^.

The specific energy and specific power of the full battery were calculated based on the mass of the active materials of both the NaTi_2_(PO_4_)_3_/C and K_0.97_Co_0.8_Mn_0.2_[Fe(CN)_6_]_0.81_•2.2H_2_O. The full cell delivers a high specific energy of 80 Wh/kg at a high specific power of 1,226.9 W/kg, which are superior to the most previous energy storage devices that use seawater as the only electrolyte (Fig. 6E) [23,48–53]. A maximum specific power can achieve 7,495 W/kg. Furthermore, the energy efficiency is 63.3% at 5 A/g, much higher than 24.0% at 3.4 A/g for the full seawater battery that we recently reported [26]. The specific energy is even comparable to the state-of-art aqueous Na^+^ batteries [10,54–56]. As a proof of concept to exhibit the potential applications, a pouch cell assembled by the K_0.97_Co_0.8_Mn_0.2_[Fe(CN)_6_]_0.81_•2.2H_2_O cathode and NaTi_2_(PO_4_)_3_/C anode was constructed. The pouch cell (5 cm × 5 cm in size) can illuminate the light-emitting diode display screen (with a rated voltage of 1 V and a rated power of 0.056 W) for about 4 min (Fig. 6F).

## Conclusion

In summary, we propose a rechargeable seawater battery that operates on a rocking-chair mechanism by using intercalation-type inorganic electrode materials. The cathode and anode materials are open-framework-type PBAs and NASICON-type NaTi_2_(PO_4_)_3_, respectively. The constructed rechargeable seawater battery can achieve simultaneously high specific energy, high specific power, greatly enhanced reversibility, and long cycle life, as well as greatly enhanced energy efficiency. The work may open new perspectives for the development of high-performance sustainable rechargeable aqueous batteries.

## Materials and Methods

The materials and methods can be found in the Supplementary Materials.

## Data Availability

All data needed to evaluate the conclusions in the paper are present in the paper and/or the Supplementary Materials.

## References

[1] Usiskin R, Lu Y, Popovic J, Law M, Balaya P, Hu YS, Maier J. Fundamentals, status and promise of sodium-based batteries. Nat Rev Mater. 2021;6:1020–1035.

[2] Lu D, Li R, Rahman MM, Yu P, Lv L, Yang S, Huang Y, Sun C, Zhang S, Zhang H, et al. Ligand-channel-enabled ultrafast Li-ion conduction. Nature. 2024;627:101–107. 38418886 10.1038/s41586-024-07045-4

[3] Yuan X, Ma F, Zuo L, Wang J, Yu N, Chen Y, Zhu Y, Huang Q, Holze R, Wu Y, et al. Latest advances in high-voltage and high-energy-density aqueous rechargeable batteries. Electrochem Energy Rev. 2021;4(1):1–34.

[4] Liu L, Du Z, Wang J, Du H, Wu S, Li M, Zhang Y, Sun J, Sun Z, Ai W. Fast-charging sodium-ion batteries enabled by molecular-level designed nitrogen and phosphorus codoped mesoporous soft carbon. Research. 2023;6:0209. 37593340 10.34133/research.0209 PMC10430870

[5] Li D, Yang S, Zheng Z, Lai WY. Constructing lithium-free anode/separator interface via 3D carbon fabric scaffold for ultrasafe lithium metal batteries. Research. 2023;6:0267. 38434242 10.34133/research.0267 PMC10907015

[6] Qiao L, Wang X, Xu R, Zhang C, Chen K, Tong K, Wang H, Dai S, Chu L, Huang M. Nitrogen-doped carbon shell armored ‘Janus’ co/Co_9_S_8_ heterojunction as robust bi-functional oxygen reduction reaction/oxygen evolution reaction catalysts in seawater-based rechargeable Zn-air batteries. Mater Today Energy. 2023;37: Article 101398.

[7] Shin J, Choi JW. Opportunities and reality of aqueous rechargeable batteries. Adv Energy Mater. 2020;10(28):2001386.

[8] Chao D, Zhou W, Xie F, Ye C, Li H, Jaroniec M, Qiao SZ. Roadmap for advanced aqueous batteries: From design of materials to applications. Sci Adv. 2020;6:eaba4098. 32494749 10.1126/sciadv.aba4098 PMC7244306

[9] Wu H, Hao J, Jiang Y, Jiao Y, Liu J, Xu X, Davey K, Wang C, Qiao SZ. Alkaline-based aqueous sodium-ion batteries for large-scale energy storage. Nat Commun. 2024;15:575. 38233408 10.1038/s41467-024-44855-6 PMC10794691

[10] Jiang L, Liu L, Yue J, Zhang Q, Zhou A, Borodin O, Suo L, Li H, Chen L, Xu K, et al. High-voltage aqueous Na-ion battery enabled by inert-cation-assisted water-in-salt electrolyte. Adv Mater. 2020;32(2):1904427. 10.1002/adma.201904427 31782981

[11] Bin D, Wang F, Tamirat AG, Suo L, Wang Y, Wang C, Xia Y. Progress in aqueous rechargeable sodium-ion batteries. Adv Energy Mater. 2018;8:1703008.

[12] Gao H, Goodenough JB. An aqueous symmetric sodium-ion battery with NASICON-structured Na_3_MnTi(PO_4_)_3_. Angew Chem Int Ed. 2016;55(41):12768–12772. 10.1002/anie.201606508 27619012

[13] Liang Z, Tian F, Yang G, Wang C. Enabling long-cycling aqueous sodium-ion batteries via Mn dissolution inhibition using sodium ferrocyanide electrolyte additive. Nat Commun. 2023;14:3591. 37328496 10.1038/s41467-023-39385-6 PMC10275921

[14] Nguyen TP, Easley AD, Kang N, Khan S, Lim SM, Rezenom YH, Wang S, Tran DK, Fan J, Letteri RA, et al. Polypeptide organic radical batteries. Nature. 2021;539:61–66. 10.1038/s41586-021-03399-1 33953410

[15] Prasad KV, Venkatakrishnan N, Mathur PB. Preliminary report on the performance characteristics of the magnesium-mercurous chloride battery system. J Power Sources. 1976;1(4):371–375.

[16] Senthilkumar ST, Go W, Han J, Thuy LPT, Kishor K, Kim Y, Y. Kim Y. Emergence of rechargeable seawater batteries. J Mater Chem A. 2019;7:22803–22825.

[17] Meng R, Zhang C, Lu Z, Xie X, Liu Y, Tang Q, Li H, Kong D, Geng CN, Jiao Y, et al. An oxygenophilic atomic dispersed Fe-N-C catalyst for lean-oxygen seawater batteries. Adv Energy Mater. 2021;11(23):2100683.

[18] Hwang SM, Park JS, Kim Y, Go W, Han J, Kim Y, Kim Y. Rechargeable seawater batteries–from concept to applications. Adv Mater. 2019;31(20):1804936. 10.1002/adma.201804936 30589114

[19] Kim Y, Künzel M, Steinle D, Dong X, Kim GT, Varzi A, Passerini S. Anode-less seawater batteries with a Na-ion conducting solid-polymer electrolyte for power to metal and metal to power energy storage. Energy Environ Sci. 2022;15:2610–2618.

[20] Kim J, Park J, Lee J, Lim WG, Jo C, Lee J. Biomass-derived P, N self-doped hard carbon as bifunctional oxygen electrocatalyst and anode material for seawater batteries. Adv Funct Mater. 2021;31(22):2010882.

[21] Li M, Dixit M, Essehli R, Jafta CJ, Amin R, Balasubramanian M, Belharouak I. Na_3_Zr_2_Si_2_PO_12_ solid electrolyte membrane for high-performance seawater battery. *Adv Sci*. 2023;**10**:2300920. 10.1002/advs.202300920 PMC10265043 37046184

[22] Nam DH, Lumley MA, Choi KS. A seawater battery with desalination capabilities enabling a dual-purpose aqueous energy storage system. Energy Stor Mater. 2021;37:556–566.

[23] Raj CJ, Manikandan R, Rajesh M, Sivakumar P, Jung H, Das SJ, Kim BC. Cornhusk mesoporous activated carbon electrodes and seawater electrolyte: The sustainable sources for assembling retainable supercapacitor module. J Power Sources. 2021;490: Article 229518.

[24] Xia QX, Shinde NM, Zhang T, Yun JM, Zhou A, Mane RS, Mathur S, Kim KH. Seawater electrolyte-mediated high volumetric MXene-based electrochemical symmetric supercapacitors. Dalton Trans. 2018;47:8676–8682. 29897071 10.1039/c8dt01375f

[25] He H, Xia Q, Wang B, Wang L, Hu Q, Zhou A. Two-dimensional vanadium carbide (V_2_CT_*x*_) MXene as supercapacitor electrode in seawater electrolyte. Chin Chem Lett. 2020;31(4):984–987.

[26] Wen W, Geng C, Li X, Li H, Wu JM, Kobayashi H, Sun T, Zhang Z, Chao D. A membrane-free rechargeable seawater battery unlocked by lattice engineering. Adv Mater. 2024;36(30): Article e2312343. 38691579 10.1002/adma.202312343

[27] Yi H, Qin R, Ding S, Wang Y, Li S, Zhao Q, Pan F. Structure and properties of Prussian blue analogues in energy storage and conversion applications. Adv Funct Mater. 2020;31(6):2006970.

[28] Pasta M, Wessells CD, Liu N, Nelson J, Mcdowell MT, Huggins RA, Toney MF, Cui Y. Full open-framework batteries for stationary energy storage. Nat Commun. 2014;5:3007. 24389854 10.1038/ncomms4007

[29] Ge J, Fan L, Rao AM, Zhou J, Lu B. Surface-substituted Prussian blue analogue cathode for sustainable potassium-ion batteries. Nat Sus. 2021;5:225–234.

[30] Jiang L, Lu Y, Zhao C, Liu L, Zhang J, Zhang Q, Shen X, Zhao J, Yu X, Li H, et al. Building aqueous K-ion batteries for energy storage. Nat Energy. 2019;4:495–503.

[31] Dong J, Lei Y, Han D, Wang H, Zhai D, Li B, Kang F. Utilizing an autogenously protective atmosphere to synthesize a Prussian white cathode with ultrahigh capacity-retention for potassium-ion batteries. Chem Commun. 2019;55:12555–12558. 10.1039/c9cc06248c 31576844

[32] Ali U, Liu B, Jia H, Li Y, Li Y, Hao Y, Zhang L, Xing S, Li L, Wang C. In situ Fe-substituted hexacyanoferrate for high-performance aqueous potassium ion batteries. Small. 2024;20(4):2305866. 10.1002/smll.202305866 37712131

[33] Yang Y, Li X, Jie B, Zheng Z, Li J, Zhu C, Wang S, Xu J, Zhang X. Electron structure modulation and bicarbonate surrounding enhance Fenton-like reactions performance of Co-Co PBA. J Hazard Mater. 2022;437: Article 129372. 35728314 10.1016/j.jhazmat.2022.129372

[34] Zeng H, Deng L, Shi Z, Luo J, Crittenden J. Heterogeneous degradation of carbamazepine by Prussian blue analogues in the interlayers of layered double hydroxides: Performance, mechanism and toxicity evaluation. J Mater Chem A. 2019;7:342–352.

[35] Li J, He L, Jiang J, Xu Z, Liu M, Liu X, Tong H, Liu Z, Qian D. Facile syntheses of bimetallic Prussian blue analogues (K_x_M[Fe(CN)_6_]·nH_2_O, M=Ni, Co, and Mn) for electrochemical determination of toxic 2-nitrophenol. Electrochim Acta. 2020;353: Article 136579.

[36] Ren L, Wang JG, Liu H, Shao M, Wei B. Metal-organic-framework-derived hollow polyhedrons of Prussian blue analogues for high power grid-scale energy storage. Electrochim Acta. 2019;321: Article 134671.

[37] Wen W, Wu J, Jiang Y, Lai L, Song J. Pseudocapacitance-enhanced Li-ion microbatteries derived by a TiN@TiO_2_ nanowire anode. Chem. 2017;2(3):404–416.

[38] Zeng Y, Lu XF, Zhang SL, Luan D, Li S, Lou XW. Construction of Co–Mn Prussian blue analog hollow spheres for efficient aqueous Zn-ion batteries. Angew Chem Int Ed. 2021;60(41):22189–22194. 10.1002/anie.202107697 PMC8518934 34313363

[39] Shang Y, Li X, Song J, Huang S, Yang Z, Xu Z, Yang HY. Unconventional Mn vacancies in Mn–Fe Prussian blue analogs: Suppressing Jahn-Teller distortion for ultrastable sodium storage. Chem. 2020;6(7):1804–1818.

[40] Mullaliu A, Gaboardi M, Plaisier JR, Passerini S, Giorgetti M. Lattice compensation to Jahn–Teller distortion in Na-rich manganese hexacyanoferrate for Li-ion storage: An operando study. ACS Appl Energy Mater. 2020;3(6):5728–5733.

[41] Wang Q, Li J, Jin H, Xin S, Gao H. Prussian-blue materials: Revealing new opportunities for rechargeable batteries. InfoMat. 2022;4(6): Article e12311.

[42] Gang P, Ping N, Yuan C, Shen L, Zhang X, Li H, Zhang C. Mesoporous NaTi_2_(PO_4_)_3_/CMK-3 nanohybrid as anode for long-life Na-ion batteries. J Mater Chem A. 2014;2:20659–20666.

[43] Venkatesha A, Seth D, Varma RM, Das S, Agarwal M, Haider MA, Bhattacharyya AJ. Probing the Na^+^/Li^+^-ions insertion mechanism in an aqueous mixed-ion rechargeable batteries with NASICON-NaTi_2_(PO_4_)_3_ anode and olivine-LiFePO_4_ cathode. ChemElectroChem. 2023;10(2): Article e202201013.

[44] Cui J, Huang T, Zhao Y, Bentley A, Xu M, Guo L, Ding M, Yang HY. Unveiling the sodium adsorption behavior of controlled NaTi_2_(PO_4_)_3_ on Ti_3_C_2_T_x_ MXene for balanced salt adsorption capacity and cycling stability. Sep Purif Technol. 2024;339: Article 126613.

[45] Man Y, Sun J, Zhao X, Duan L, Fei Y, Bao J, Mo X, Zhou X. An ultrastable sodium-ion battery anode enabled by carbon-coated porous NaTi_2_(PO_4_)_3_ olive-like nanospheres. J Colloid Interface Sci. 2023;635:417–426. 36599240 10.1016/j.jcis.2022.12.155

[46] Roh HK, Kim HK, Kim MS, Kim DH, Chung KY, Roh KC, Kim KB. In situ synthesis of chemically bonded NaTi_2_(PO_4_)_3_/rGO 2D nanocomposite for high-rate sodium-ion batteries. Nano Res. 2016;9:1844–1855.

[47] He B, Yin K, Gong W, Xiong Y, Zhang Q, Yang J, Wang Z, Wang Z, Chen M, Man P, et al. NaTi_2_(PO_4_)_3_ hollow nanoparticles encapsulated in carbon nanofibers as novel anodes for flexible aqueous rechargeable sodium-ion batteries. Nano Energy. 2021;82: Article 105764.

[48] Nimkar A, Gavriel B, Bergman G, Turgeman M, Fan T, Shpigel N, Aurbach D. Rechargeable seawater batteries based on polyimide anodes. ACS Sustain Chem Eng. 2023;11(4):1428–1433.

[49] Liu Y, Luo L, Shen Z, Ji Y, Wen Z, Li Z, Li J, Sun P, Xie J, Hong G. Bipolar Prussian blue analogues electrodes for symmetric aqueous batteries in diverse scenarios. Chem Eng J. 2023;465: Article 142733.

[50] Vijay CJ, Vijayakumar M, Rohita DS, Elsa G, Sankar AB, Rao TN, Karthik M. Hierarchical activated carbon fibers as a sustainable electrode and natural seawater as a sustainable electrolyte for high-performance supercapacitor. Energ Technol. 2020;8(9):2000417.

[51] Zhang B, Zhang C, Yuan W, Yang O, Liu Y, He L, Hu Y, Zhou L, Wang J, Wang ZL. Highly stable and eco-friendly marine self-charging power systems composed of conductive polymer supercapacitors with seawater as an electrolyte. ACS Appl Mater. 2022;14(7):9046–9056. 10.1021/acsami.1c22129 35143173

[52] Zhao P, Yao M, Zhang Q, Wang N, Hu W, Komarneni S. Electrochemical behavior of representative electrode materials in artificial seawater for fabricating supercapacitors. Electrochim Acta. 2019;318:211–219.

[53] Cheng S, Dai Z, Fu J, Cui P, Wei K, Zhang Y, Wu Y, Liu Y, Sun Z, Shao Z, et al. Towards large-scale electrochemical energy storage in the marine environment with a highly-extensible “paper-like” seawater supercapacitor device. J Mater Chem A. 2021;9:622–631.

[54] Meng QY, Shao JC, Dou XR, Chi HZ. N-containing Na_2_VTi(PO_4_)_3_/C for aqueous sodium-ion batteries. Small. 2024;2308483. 10.1002/smll.202308483 38329171

[55] Hou Z, Zhang X, Chen J, Qian Y, Chen LF, Lee P. Towards high-performance aqueous sodium ion batteries: Constructing hollow NaTi_2_(PO_4_)_3_@C nanocube anode with Zn metal-induced pre-sodiation and deep eutectic electrolyte. Adv Energy Mater. 2022;12(14):2104053.

[56] Shan X, Guo F, Charles DS, Lebens-Higgins Z, Razek S, Wu J, Xu W, Yang W, Page KL, Neuefeind JC, et al. Structural water and disordered structure promote aqueous sodium-ion energy storage in sodium-birnessite. Nat Commun. 2019;10:4975. 31672984 10.1038/s41467-019-12939-3 PMC6823464

