# Supplementary material for: A Rocking-chair Rechargeable Seawater Battery
Source: Research (Wash D C). 2024 Aug 27;7:0461. doi: 10.34133/research.0461 (PMC11347753; doi:10.34133/research.0461)
Supplement: Supplementary 1 — Figs. S1 to S16 Tables S1 to S3 [file research.0461.f1.pdf]

Supporting Information

A Rocking-Chair Rechargeable Seawater Battery

Jialong Wu,[a] Yongshuo Zheng,[a] Pengfei Zhang,[a] Xiaoshuang Rao,[a] Zhenyu Zhang,[a,b] Jin-Ming Wu,[c] and Wei Wen[a*]

[a]Collaborative Innovation Center of Ecological Civilization, School of Mechanical and Electrical Engineering, Hainan University, Haikou 570228, China.

[b]State Key Laboratory of High-performance Precision Manufacturing, Dalian University of Technology, Dalian 116024, China.

[c]State Key Laboratory of Silicon and Advanced Semiconductor Materials, School of Materials Science and Engineering, Zhejiang University, Hangzhou 310027, China.

*Corresponding author, E-mail: wwen@hainanu.edu.cn

Materials and Methods

Material Synthesis. For the synthesis of the $K_{0.97}Co_{0.8}Mn_{0.2}[Fe(CN)_6]_{0.81}\cdot 2.2H_2O$, $CoCl_2\cdot 6H_2O$ (0.008 mol), $MnCl_2$ $4H_2O$ (0.002 mol), and sodium citrate (0.01 mol) were dissolved in 50 mL of deionized water. Together with a 0.01 mol of $K_3[Fe(CN)_6]$ aqueous solution (50 mL), the above solution was slowly added to 100 ml of deionized water and the reaction was conducted for 12 h at room temperature, resulting in the formation of a lilac precipitate. Finally, the precipitate was washed and then dried in an oven to obtain the sample. To investigate the effect of the Co/Mn ratio, the proportions of $CoCl_2$ and $MnCl_2$ in the preparation were adjusted, without the addition of sodium citrate. The synthesis processes of the $NaTi_2(PO_4)_3$/C were adopted from the previous literature [55].

Characterizations. XRD measurements were conducted using a SmartLab X-ray diffractometer (Rigaku corporation, Japan) with Cu Kα radiation at 20 kV and 10 mA (λ =0.15406 nm). For the Rietveld refinement, the XRD tests were carried out on a Bruker D8 Advance diffractometer with a step size of 0.02^{o} and time/step of 0.2 s, operated at 40 kV and 40 mA. The morphologies of the materials were observed by a field emission scanning electron microscopy (FESEM, Verios G4 UC), equipped with an energy dispersive X-ray spectroscopy (EDX), and a transmission electron microscopy (TEM, Talos F200S) at 200 kV. The Brunauer-Emmett-Teller (BET) approach using adsorption data was utilized to determine the specific surface areas of the cathode and anode materials. The samples were degassed at 150 °C for 8 h to remove physisorbed gases prior to the low-temperature nitrogen adsorption/desorption measurements on an ASAP 2020. The X-ray photoelectron spectra (XPS) characterizations were conducted on a Thermo Scientific K-ALPHA with Al Kα radiation (h*ν* = 1486.8 eV) working at 150 W and 15.0 kV. All the binding energies for XPS measurements were calibrated *via* using the containment carbon at 284.8 eV. Raman measurements were carried out on an inVia Raman Microscope (Renishaw plc) with a Nd:YAG intracavity doubled laser operated at 514 nm. FTIR tests were conducted on a NICOLET iS50 FT-IR. ICP-OES analysis was carried out on a Plasma 3000.

Electrochemical Measurements. A slurry coating process was utilized for the preparation of the working electrodes. The active material, acetylene black, and polyvinylidene fluoride (PVDF) were mixed at a ratio of 7:2:1 in the slurry, which was dispersed in N-methyl pyrrolidinone (NMP) and then coated onto a carbon paper current collector (2 cm×2 cm in size). For XRD analysis, carbon

cloth was also used as current collector, because the diffraction intensity of carbon in carbon cloth is much lower than that in carbon paper, which is beneficial for observing diffraction peaks of the electrode materials. The mass loadings of the active materials for cathode and anode were 1-2 mg/cm^2. The obtained films were dried at 60 ^oC for 12 h. A CHI660E electrochemical workstation (Chenhua, China) and a CT-3002A Landt battery test system were used for the electrochemical performance measurements. All the measurements were conducted at room temperature. The natural seawater was taken from the west coast of Haikou city, China, naturally settled to remove the suspended particles, and used as the electrolyte for half-cells and full-cells without any other treatment. The performances of half-cells were tested by a standard three-electrode system, in which the cathode or anode materials were used as working electrode. Pt electrode and Ag/AgCl electrode acted as counter electrode and reference electrode, respectively. For the full-cells, the $K_{0.97}Co_{0.8}Mn_{0.2}[Fe(CN)_6]_{0.81}•2.2H_2O$ and $NaTi_2(PO_4)_3$/C were used as the active materials for the cathode and anode, respectively.

The specific capacity (Q, mAh/g) was calculated based on the GCD curves as follows:

$$Q = \frac{I \times t}{3.6m} \tag{1}$$

where I represent the current (A), t is the time (s) of the deintercalation (oxidation process) for anode or the intercalation (reduction process) for cathode, and m means the weight (g) of the active material in the cathode or anode. The factor of 3.6 was used for the unit conversion from C to mAh.

For the full cells, specific energy E and specific power P were calculated by the following equations based on GCD curves:

$$E = \frac{I\int U\mathrm{d}t}{m_{C+A}} \tag{2}$$

$$P = \frac{E}{t} \tag{3}$$

where U, I, and t express voltage, current, and discharge time, respectively. The m_{C+A} represents the mass of the active materials of the $K_{0.97}Co_{0.8}Mn_{0.2}[Fe(CN)_6]_{0.81} • 2.2H_2O$ cathode and the $NaTi_2(PO_4)_3$/C anode.

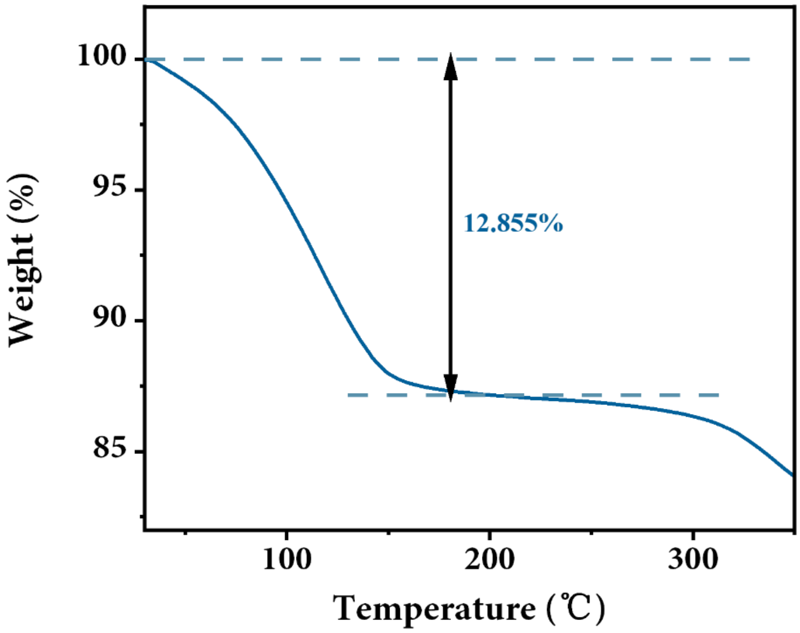

Figure S1. TG curve of $K_{0.97}Co_{0.8}Mn_{0.2}[Fe(CN)_6]_{0.81}$•$2.2H_2O$ cathode material.

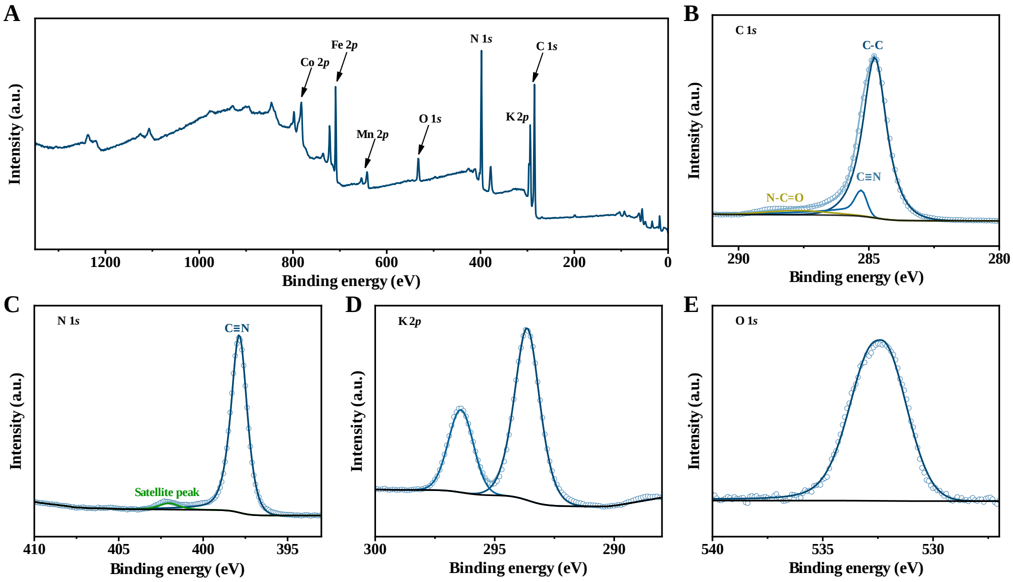

Figure S2. Survey XPS spectrum and high-resolution XPS spectra of $K_{0.97}Co_{0.8}Mn_{0.2}[Fe(CN)_6]_{0.81}$• $2.2H_2O$ cathode material. (A) Survey spectrum, (B) C 1*s* spectrum, (C) N 1*s* spectrum, (D) K 2*p* spectrum, and (E) O 1*s* spectrum.

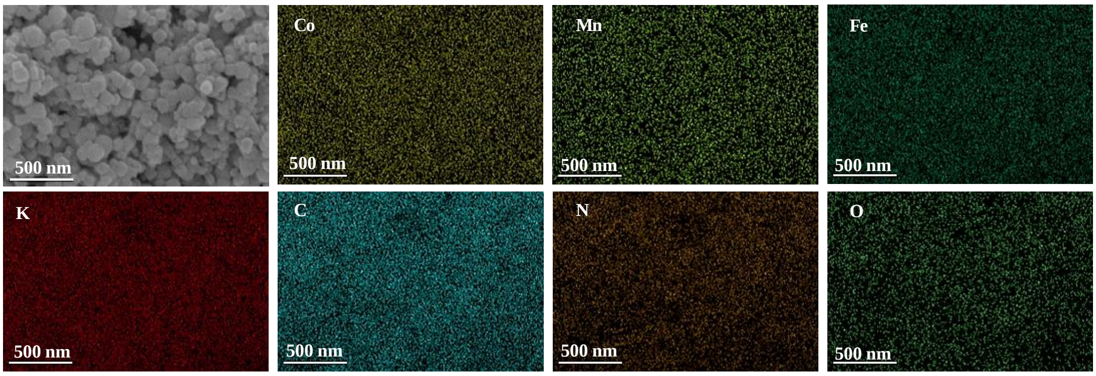

Figure S3. SEM image and EDX mapping of $K_{0.97}Co_{0.8}Mn_{0.2}[Fe(CN)_6]_{0.81}$•$2.2H_2O$ cathode material.

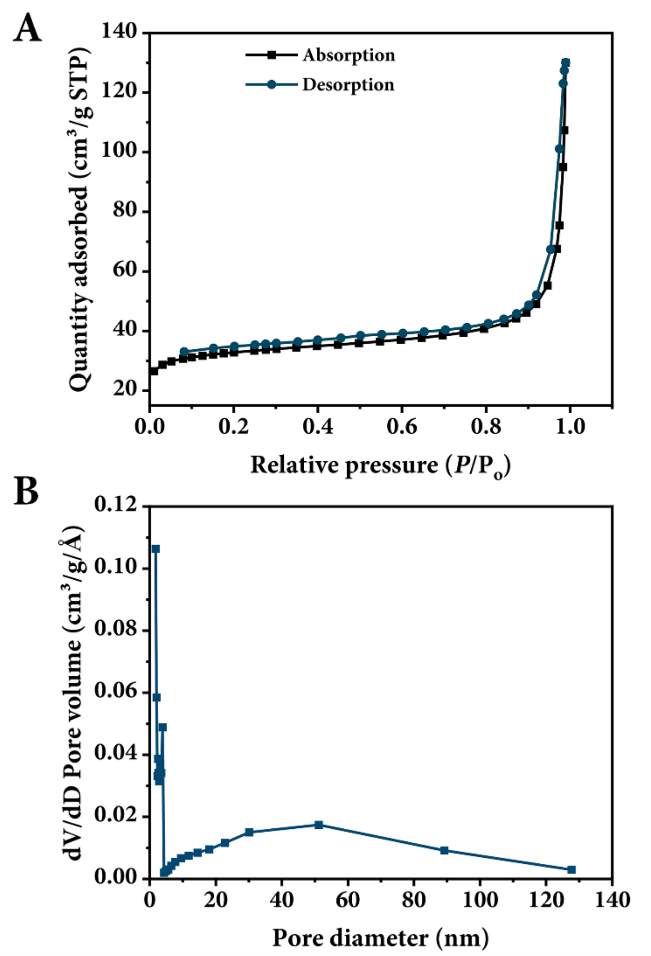

Figure S4. (A) N_2 adsorption-desorption isotherm curves and (B) pore size distribution of $K_{0.97}Co_{0.8}Mn_{0.2}[Fe(CN)_6]_{0.81}\cdot 2.2H_2O$ cathode material.

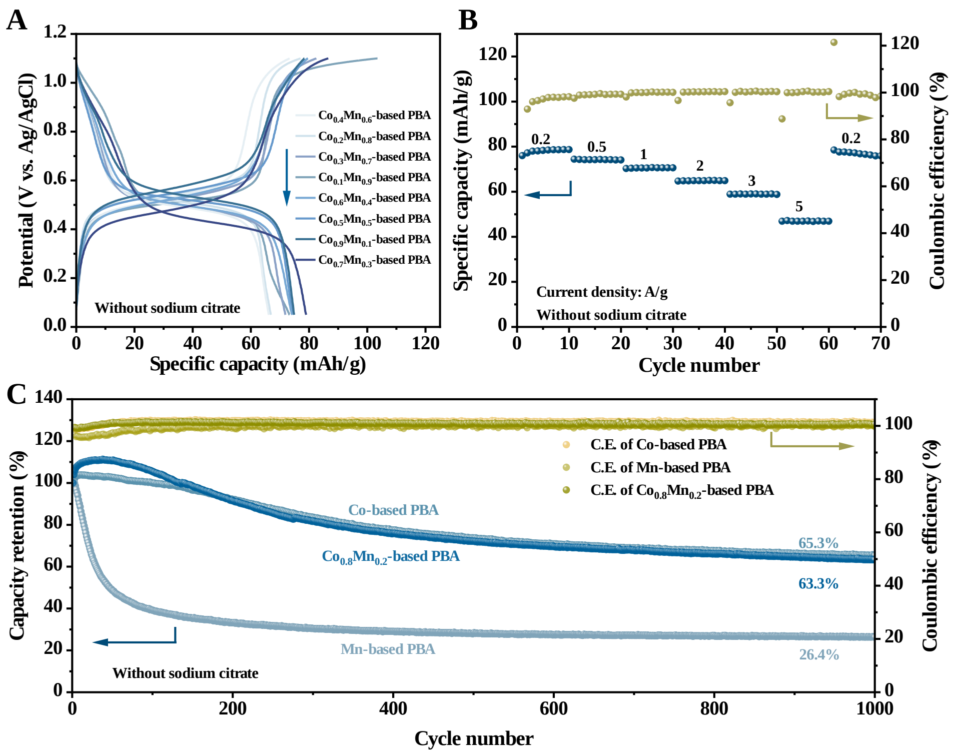

Figure S5. (A) GCD curves of $Co_{0.9}Mn_{0.1}$-based PBA, $Co_{0.7}Mn_{0.1}$-based PBA, $Co_{0.6}Mn_{0.4}$-based PBA, $Co_{0.5}Mn_{0.5}$-based PBA, $Co_{0.4}Mn_{0.6}$-based PBA, $Co_{0.3}Mn_{0.7}$-based PBA, $Co_{0.2}Mn_{0.8}$-based PBA, $Co_{0.1}Mn_{0.9}$-based PBA at 0.5 A/g. All the samples were prepared without the addition of sodium citrate. (B) Rate performance of $Co_{0.8}Mn_{0.2}$-based PBA sample, which was prepared without the addition of sodium citrate. (C) Cycling stability of Mn-based PBA, Co-based PBA and $Co_{0.8}Mn_{0.2}$-based PBA at 3 A/g. All the samples were prepared without the addition of sodium citrate.

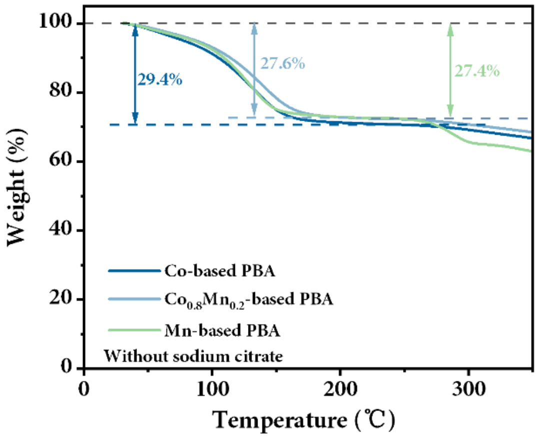

Figure S6. TG curves for Mn-based PBA, Co-based PBA and $Co_{0.8}Mn_{0.2}$-based PBA samples. All the samples were prepared without the addition of sodium citrate.

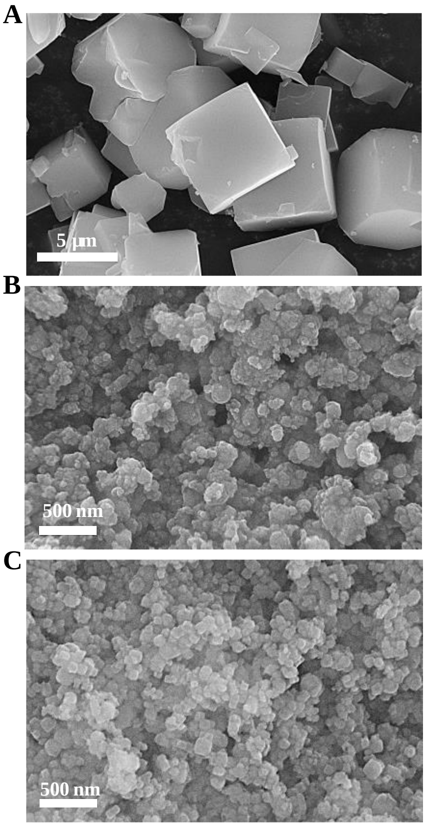

Figure S7. SEM images of (A) Mn-based PBA, (B) Co-based PBA and (C) $Co_{0.8}Mn_{0.2}$-based PBA samples. All the samples were prepared without the addition of sodium citrate.

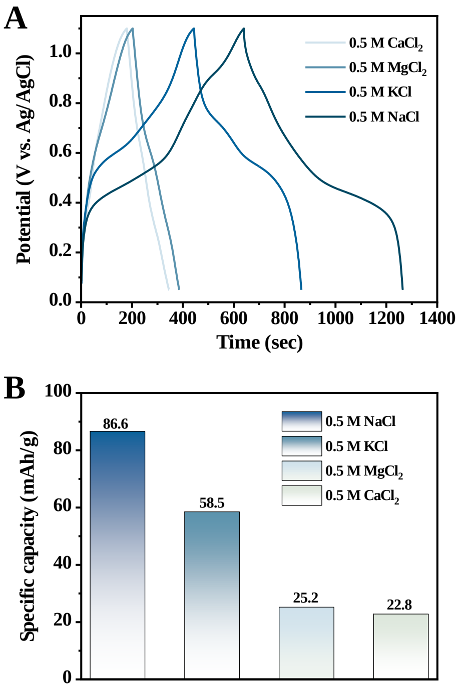

Figure S8. (A) GCD curves at 0.5 A/g in different electrolytes and (B) the specific capacities of $K_{0.97}Co_{0.8}Mn_{0.2}[Fe(CN)_6]_{0.81}$•$2.2H_2O$ cathode material.

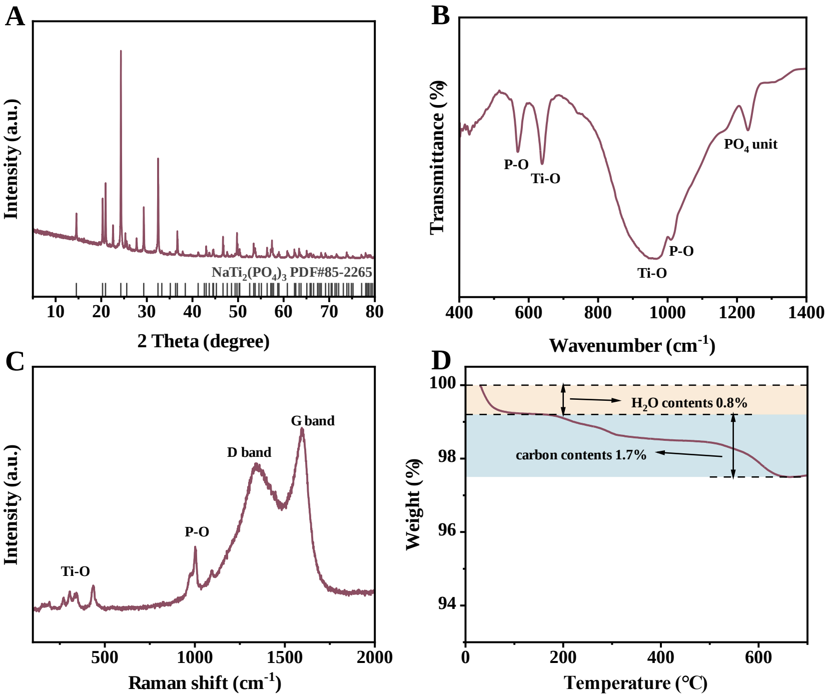

Figure S9. (A) XRD patterns, (B) FTIR spectrum, (C) Raman spectrum, and (D) TG curve of $NaTi_2(PO_4)_3$/C anode material.

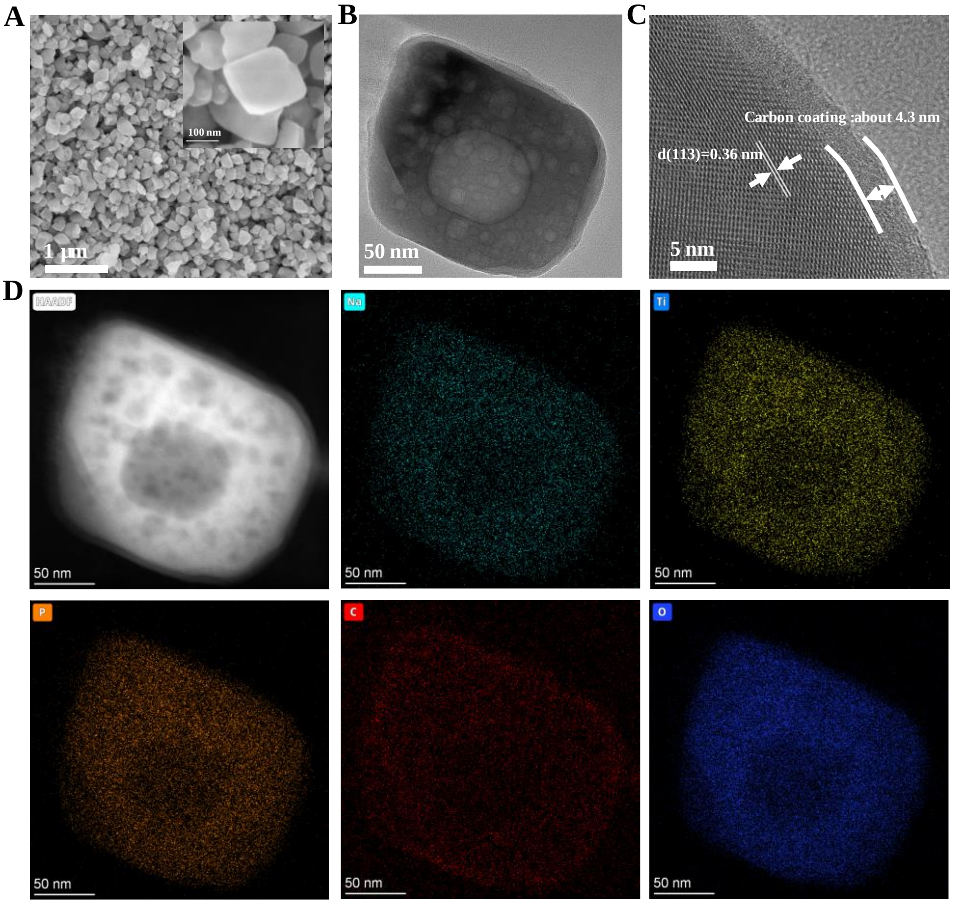

Figure S10. (A) SEM image, (B) TEM image, (C) HRTEM image, and (D) EDX mapping of $NaTi_2(PO_4)_3$/C anode material.

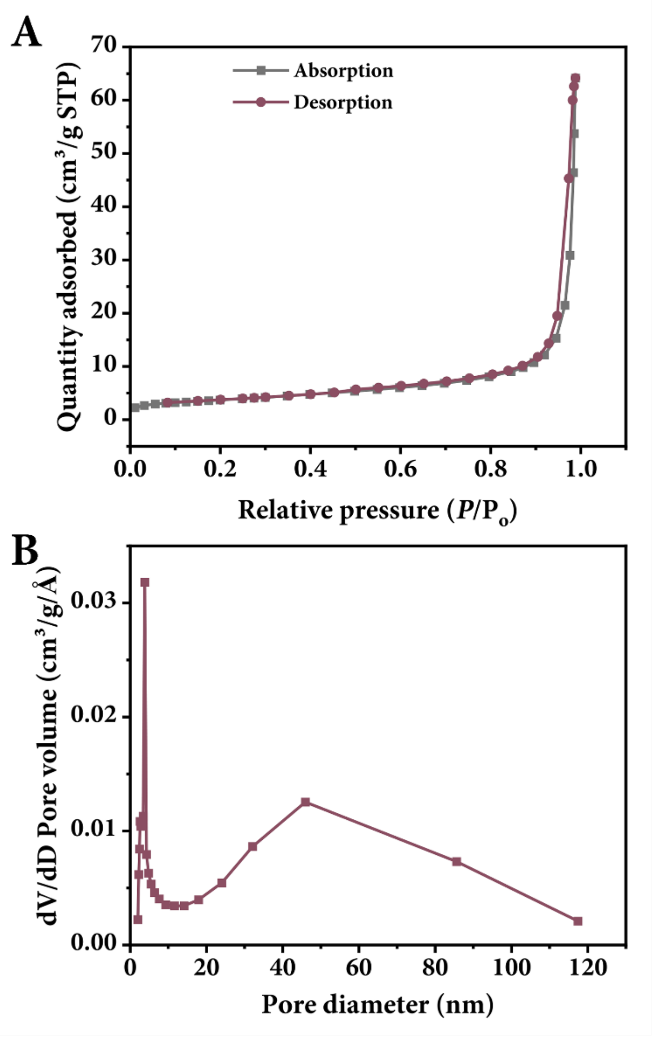

Figure S11. (A) N_2 adsorption-desorption isotherm curves and (B) pore size distribution of $NaTi_2(PO_4)_3$/C anode material.

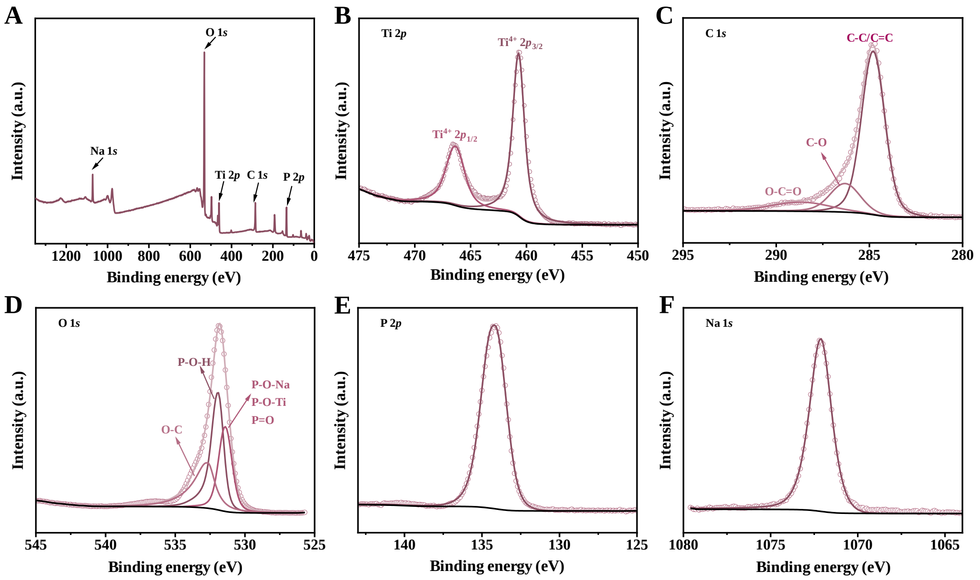

Figure S12. Survey XPS spectrum and high-resolution XPS spectra of $NaTi_2(PO_4)_3$/C anode material. (A) Survey XPS spectrum. (B) Ti 2*p* spectrum, (C) C 1*s* spectrum, (D) O 1*s* spectrum, (E) P 2*p* spectrum, (f) Na 1*s* spectrum.

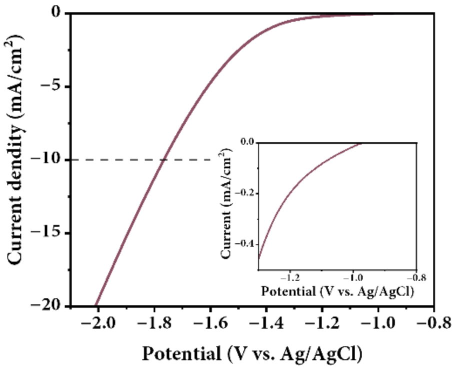

Figure S13. LSV polarization curve of $NaTi_2(PO_4)_3$/C anode material at 5 mV/s. The inset shows the locally enlarged image.

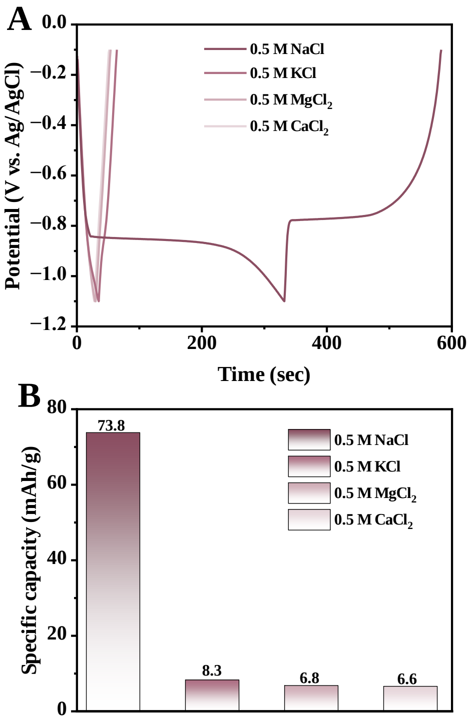

Figure S14. (A) GCD curves at 1.1 A/g in different electrolytes and (B) the specific capacities of $NaTi_2(PO_4)_3$/C anode material.

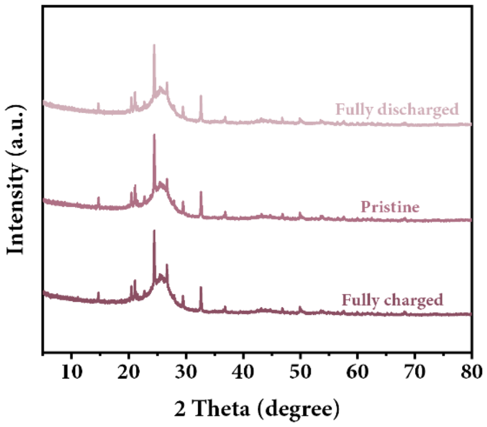

Figure S15. XRD patterns of $NaTi_2(PO_4)_3$/C anode material at the pristine and fully charged/discharged states.

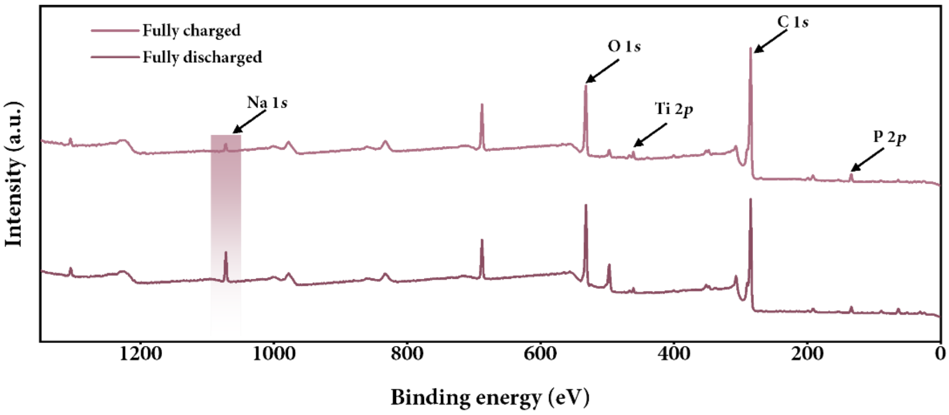

Figure S16. Survey XPS spectra of $NaTi_2(PO_4)_3$/C anode material at fully charged and discharged states.

Table S1. Relative atomic ratios of different elements to the total amount of Co and Mn in the $K_{0.97}Co_{0.8}Mn_{0.2}[Fe(CN)_6]_{0.81}\cdot2.2H_2O$ sample at charge/discharge states in different cycles.

Element	Pristine	Charged	Discharged	200 cycles discharged	500 cycles discharged
**Na**	0	0.02	0.47	0.51	0.85
**K**	0.97	0.18	0.6	0.65	0.38
**Ca**	0	0	0.01	0.06	0.1
**Mg**	0	0.04	0.04	0.28	0.28

The results were obtained from EDX characterizations and the total amount of Co and Mn serves as the reference element (normalized to 1) for the ratios.

Table S2. Relative atomic ratios of different elements to Ti in the $NaTi_2(PO_4)_3/C$ sample at charge/discharge states in different cycles.

Element	Pristine	Charged	Discharged
**Na**	0.5	0.67	1.3
**K**	0	0	0
**Ca**	0	0	0.08
**Mg**	0	0.11	0.1
**P**	1.5	1.45	1.52

The results were obtained from EDX characterizations and the amount of Ti serves as the reference element (normalized to 1) for the ratios.

Table S3. The comparison of specific energy and specific power for seawater batteries and aqueous Na^+ batteries.

Aqueous battery type	Anode	Cathode	Specific energy (Wh/kg)	Specific power (W/kg)	Ref.
**Seawater batteries**	**$NaTi_2(PO_4)_3$**	**$K_{0.97}Co_{0.8}Mn_{0.2}[Fe(CN)_6]_{0.81}\cdot 2.2H_2O$**	**80<br>23.7**	**1226.9<br>7495**	**This work**
Seawater batteries	TiO_2	$Co_xMn_{3-x}O_4$	102.5<br>86.4	1362.5<br>1574.9	[26]
Seawater supercapacitors	CHAC	CHAC	7.4	6330	[23]
Seawater batteries	PI	Ni-HCF	≈ 27.1	≈ 862	[48]
Seawater batteries	FeHCF	FeHCF	≈ 17.5	≈ 30.1	[49]
Seawater supercapacitors	ACF	ACF	≈ 3.5<br>≈ 2.5<br>≈ 1	≈ 100<br>≈ 1000<br>≈ 3000	[50]
Seawater supercapacitors	h-PPy	h-PPy	5.12	4320	[51]
Seawater supercapacitors	PGNPC	PMNR	20.4	781	[52]
Seawater supercapacitors	Fe_2O_3@CNFs	MnO_2@CNTs	43.1	667	[53]
Na^+ batteries	$NaTiOPO_4$	$Na_{1.88}Mn[Fe(CN)_6]_{0.97}$	71	62	[10]
Na^+ batteries	N-NaVTP	N-NaVTP	55.9	225	[54]
Na^+ batteries	$NaTi_2(PO_4)_3$@C	$Na_{0.44}MnO_2$	50	10	[55]
Na^+ batteries	$Na_{0.27}MnO_2$	$Na_{0.27}MnO_2$	28	600	[56]

All the specific energies were calculated based on the total mass of active materials in the anode and cathode.
